# Clinical scoring system to differentiate melioidosis from other documented causes of community-acquired bacterial pneumonia: a retrospective cohort study

**DOI:** 10.1007/s15010-025-02611-y

**Published:** 2025-07-23

**Authors:** Nitin Gupta, Chiranjay Mukhopadhyay, Tirlangi Praveen Kumar, Kavita Salian, Prithvishree Ravindra, Rachana Bhat, Steven Van Den Broucke, Emmanuel Bottieau, Erika Vlieghe

**Affiliations:** 1 Department of Infectious Diseases, Kasturba Medical College, Manipal, Manipal Academy of Higher Education, Manipal, 576104 India; 2 Department of Clinical Sciences, Institute of Tropical Medicine, Antwerp, Belgium; 3 University of Antwerp, Antwerp, Belgium; 4 Department of Microbiology, Kasturba Medical College, Manipal, Manipal Academy of Higher Education, Manipal, 576104 India; 5 Department of Emergency Medicine, Kasturba Medical College, Manipal, Manipal Academy of Higher Education, Manipal, 576104 India; 6 University Hospital Antwerp, Antwerp, Belgium

**Keywords:** Melioidosis, Community-acquired pneumonia, Clinical scoring system, Diabetes mellitus, Monsoon season

## Abstract

**Background:**

Melioidosis, caused by *Burkholderia pseudomallei*, is an underdiagnosed cause of community-acquired pneumonia (CAP) in India. Due to overlapping features with other bacterial pneumonias and limited access to culture facilities, early diagnosis and treatment remain challenging. This study aimed to develop a clinical scoring system to distinguish melioidosis from other bacterial causes of CAP in an endemic setting.

**Methods:**

We conducted a retrospective cohort study of 337 patients with radiologically confirmed blood or respiratory culture-positive CAP cases at a tertiary care hospital in South India from 2017 to 2023. This included 55 melioidosis cases and 282 controls with other documented bacterial etiologies. Demographic, clinical, laboratory, and radiological variables were compared. Multivariable logistic regression identified independent predictors of melioidosis. A scoring system was developed using the natural logarithms of adjusted odds ratios (aORs).

**Results:**

Four independent predictors were retained in the final model: monsoon season exposure (aOR = 9.0, 95% CI: 3.6–22.6), diabetes mellitus (aOR = 10.1, 95% CI: 3.6–28.5), shock at presentation (aOR = 17.2, 95% CI: 5.9–49.9), and extrapulmonary focal involvement (aOR = 36.5, 95% CI: 11.0–121.4). The model showed excellent discrimination. A score of ≥ 4 out of 11 yielded a sensitivity of 87.3% and specificity of 83.6%, while a score of ≥ 5 yielded a sensitivity and specificity of 67.3% and 95.4%, respectively.

**Conclusion:**

We propose a simple four-point clinical scoring tool to identify melioidosis in patients with CAP. This score can guide early suspicion and appropriate therapy in endemic resource-limited settings. Prospective validation in other endemic regions is warranted.

**Supplementary Information:**

The online version contains supplementary material available at 10.1007/s15010-025-02611-y.

## Introduction

Community-acquired pneumonia (CAP) remains a leading cause of hospital admissions and antibiotic use worldwide [1]. CAP is a significant health burden in India, accounting for 23% of global cases [2]. The empirical treatment for CAP requiring admission typically involves β-lactams like ceftriaxone or amoxicillin-clavulanate with or without a macrolide such as azithromycin [3]. However, these regimens offer inadequate coverage against *Burkholderia pseudomallei*, the causative agent of melioidosis, a life-threatening infection endemic to several tropical regions [4, 5]. It is increasingly recognised in the monsoon months in South Asia [6]. Although melioidosis is a multisystem disease, it most commonly presents as pneumonia [7]. In one of the largest cohorts of melioidosis, pneumonia as the presenting feature was noted in over half of the patients [8].

The disease is underdiagnosed due to clinical unfamiliarity and limited diagnostic infrastructure in endemic regions [6]. The diagnosis of melioidosis relies on culture-based methods that require prolonged incubation and may not be widely available in peripheral health facilities [4]. This lack of access to timely and accurate diagnostics often forces clinicians to rely heavily on subjective clinical judgment. Melioidosis may present in acute, subacute, or chronic forms. Still, the acute presentation is particularly associated with higher mortality and demands a high index of suspicion for timely initiation of appropriate therapy. It is often challenging to differentiate it from other common bacterial infections, such as *Streptococcus pneumoniae*, *Haemophilus influenzae*, *Staphylococcus aureus*, and *Klebsiella pneumoniae*, especially without structured, evidence-based guidance. As a result, primary care physicians may struggle to recognise melioidosis promptly, leading to potential delays in diagnosis and treatment [9]. This diagnostic uncertainty can result in delayed or missed diagnoses, resulting in poor outcomes and the overuse of multiple broad-spectrum empiric antibiotics, further selecting out antimicrobial resistance. Without rapid confirmatory tests and the lack of blood culture facilities in many settings, a validated clinical scoring system may help guide frontline healthcare providers toward earlier suspicion of melioidosis and appropriate management. This study, therefore, aims to identify key clinical predictors that distinguish melioidosis from other common bacterial causes of CAP and to develop a simple, easy-to-use clinical scoring system for use in similar hospital-based settings.

## Methodology

### Study design and setting

This retrospective cohort study was conducted at a tertiary care hospital in coastal southern India. Hospital records were screened for all adult patients who presented between January 1, 2017, and December 31, 2023, and met the eligibility criteria. This study was approved by the Institutional Ethics Committee of Kasturba Medical College and Kasturba Hospital, Manipal (IEC1: 430/2023).

### Case definition and eligibility criteria

CAP was defined as the presence of a presumed new infiltrate on chest X-Ray (CXR), along with at least two of the following clinical features: fever (≥ 38 °C), cough, or dyspnoea (either self-reported or documented respiratory distress), with symptom duration of less than 15 days. CAP with bacterial cultural positivity from a respiratory specimen (e.g., sputum, bronchoalveolar lavage, endotracheal aspirate) or a blood specimen collected within two days of hospital admission was included. Patients were excluded if they had no radiological infiltrates, hospital-acquired or polymicrobial infections, symptoms > 14 days, or isolates uncommon in CAP (e.g., *Pseudomonas*, *Acinetobacter*, *Stenotrophomonas*) (Supplementary Table 1).

### Sample size estimation

Based on a prior study from an endemic setting indicating that approximately 11% of CAP cases in similar settings are due to melioidosis, and applying the rule of at least seven outcome events per predictor variable in logistic regression, a minimum of four independent variables anticipated in the final model and allowing a 25% buffer for missing data, the estimated sample size required for this study was 340 patients with CAP [10, 11].

### Case and control definitions

Patients fulfilling the CAP case definition with culture-confirmed *Burkholderia pseudomallei* infection were defined as melioidosis cases. Patients meeting the same clinical and radiological criteria but with other documented bacterial etiologies were included as controls.

### Data collection

Demographic, clinical, laboratory, and radiological data were extracted from electronic medical records. Variables included age, sex, monsoon season presentation, comorbidities (pre-existing or newly diagnosed), lifestyle factors (alcohol use, smoking), and key clinical features at presentation. Laboratory parameters and radiological findings were also collected. Where appropriate, categorical thresholds were applied to improve interpretability. Definitions of clinical and laboratory variables used in the analysis are detailed in Supplementary Table 2.

### Statistical analysis

Descriptive statistics were used to summarise and compare demographic, clinical, laboratory, and radiological features between melioidosis cases and controls. Categorical variables were compared using the Chi-square test. In contrast, continuous variables were assessed using the Student’s t-test or the Mann-Whitney U test, based on the data distribution. A two-tailed *p-*value < 0.05 was considered statistically significant. Variables that were statistically significant on univariate analysis, demonstrated a positive association with melioidosis, and were considered clinically relevant were selected for inclusion in the final logistic regression model. Variables with a high proportion of missing data (e.g., CRP) or those not routinely available in peripheral settings (e.g., AST, ALT) were excluded to enhance the model’s applicability. To address multicollinearity, only one variable from highly correlated pairs was retained. For instance, between bilirubin levels and the clinical finding of icterus, icterus was included in the model. Variables with a *p*-value < 0.05 on univariate analysis and those of established clinical relevance were included in a multivariable logistic regression model to identify independent predictors of melioidosis. Adjusted odds ratios (aORs) with 95% confidence intervals were calculated.

### Development of the scoring system

A clinical scoring system was developed using the aORs from the final logistic regression model. Each aOR was converted to its natural logarithmic value and rounded to the nearest whole number to assign a score weight. A composite score was calculated for each patient, and a receiver operating characteristic (ROC) curve was generated to determine the Area Under the Curve (AUC) and the optimal cut-off point that maximised sensitivity and specificity for diagnosing melioidosis among patients with CAP.

### Calculation of the likelihood ratios of individual components of the scoring system

To assess the diagnostic performance of each predictor included in the melioidosis scoring system, we conducted univariate ROC curve analyses. AUC, sensitivity, and specificity were calculated for each variable using the binary classification of melioidosis as the outcome. Using standard formulas, positive and negative likelihood ratios (LR+⁺ and LR⁻) were computed from sensitivity and specificity estimates.

### Comparison of scoring system variables amongst the microbial causes

To examine how the clinical predictors included in the scoring system vary across different microbial causes of CAP, we conducted cross-tabulation analyses. These variables were selected based on their inclusion in the final diagnostic scoring model for melioidosis.

## Results

**Fig. 1:**
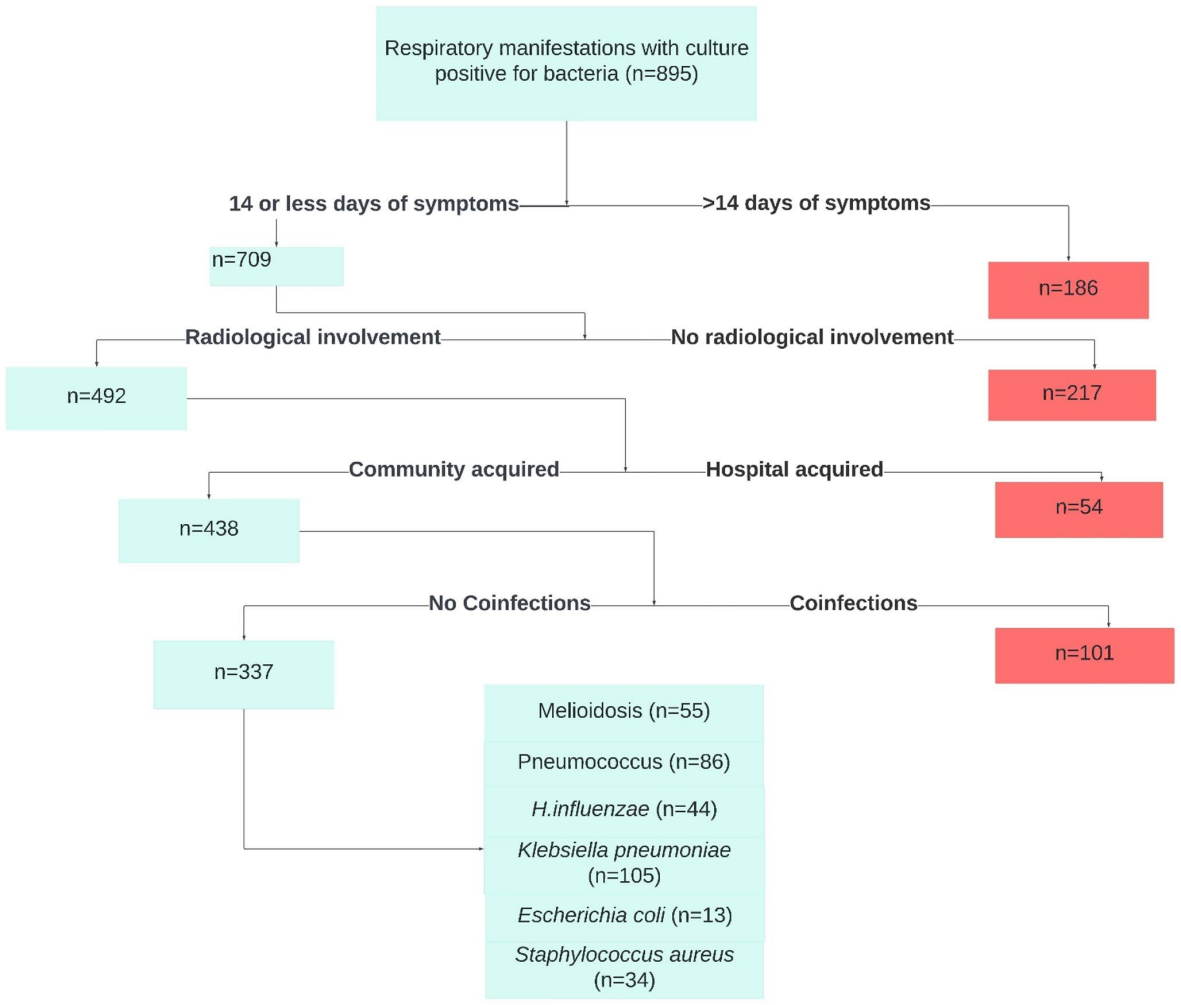
Flowchart showing the selection of patients with community-acquired bacterial pneumonia included in the final analysis. Patients with bacterial respiratory infections were screened. Exclusions (red) included prolonged symptoms, no radiological findings, hospital-acquired infection, or coinfections. The final included cases (n=337) with community-acquired pneumonia and single-organism infection are represented in green

Of 895 patients presenting with respiratory manifestations and culture-confirmed bacterial infection, 709 had ≤ 14 days of symptoms. After excluding patients with no radiological involvement (*n* = 217), hospital-acquired infections (*n* = 54), and coinfections (*n* = 101), 337 patients with bacterial CAP and no coinfections were included. None of the excluded patients with coinfections had melioidosis. The final cohort comprised patients infected with *Burkholderia pseudomallei* (melioidosis, *n* = 55), *Streptococcus pneumoniae* (*n* = 86), *Haemophilus influenzae* (*n* = 44), *Klebsiella pneumoniae* (*n* = 105), *Escherichia coli* (*n* = 13), and *Staphylococcus aureus* (*n* = 34) (Fig. 1). Melioidosis was considered as ‘case’, and the rest were considered as controls.

Melioidosis cases were more likely to occur during the monsoon season in older male patients with diabetes and poorer glycaemic control (Table 1). They more frequently presented with shock, lymphadenopathy, altered sensorium, icterus, and extrapulmonary abscesses. Laboratory abnormalities such as lymphopenia, thrombocytopenia, hypoalbuminemia, and raised creatinine, urea, transaminases, and CRP were significantly more common. Radiologically, bilateral infiltrates were more frequent. Bacteremia (80% vs. 12.9%) and 14-day mortality (34.5% vs. 3.2%) were also significantly higher in melioidosis cases (all *p* < 0.001).

**Table 1:** Comparison of demographic, clinical, laboratory, radiological, and outcome parameters between cases (Melioidosis) and controls (Other Community-Acquired Pneumonia)

Variable	Cases (*n* = 55)	Controls (*n* = 282)	*p*-value
**Presentation in Monsoon (June to September)**	**41/55 (74.5%)**	**70/282 (24.8%)**	**< 0.001**
**Male gender**	**42/55 (76.4%)**	**176/282 (62.4%)**	**0.048**
**Age (years)**	**53.18 ± 12.77**	**58.47 ± 14.46**	**0.012**
**Diabetes Mellitus**	**46/55 (83.6%)**	**106/280 (37.9%)**	**< 0.001**
**HbA1c (%)**	**10.17 ± 3.22 (** *n* ** = 50)**	**7.03 ± 2.15 (** *n* ** = 195)**	**< 0.001**
Alcohol Use	6/55 (10.9%)	15/282 (5.3%)	0.117
Smoking	4/55 (7.3%)	17/282 (6.0%)	0.727
Malignancy	2/55 (3.6%)	23/282 (8.2%)	0.242
HIV	0/55 (0%)	4/282 (1.4%)	0.374
CKD	3/55 (5.5%)	18/282 (6.4%)	0.794
CLD	1/55 (1.8%)	10/282 (3.5%)	0.509
**Chronic Lung Disease**	**8/55 (14.5%)**	**119/282 (42.2%)**	**< 0.001**
Diarrhoea	3/53 (5.7%)	6/282 (2.1%)	0.144
**Icterus**	**13/55 (23.6%)**	**10/282 (3.5%)**	**< 0.001**
**Lymphadenopathy**	**8/55 (14.5%)**	**5/282 (1.8%)**	**< 0.001**
**Shock**	**24/55 (43.6%)**	**14/282 (5.0%)**	**< 0.001**
**Altered sensorium**	**7/55 (12.7%)**	**11/282 (3.9%)**	**0.008**
Neck signs	1/55 (1.8%)	2/282 (0.7%)	0.423
**Extrapulmonary focal abscesses**	**20/55 (36.3%)**	**11/282 (3.9%)**	**< 0.001**
Total Leukocyte Count (×10⁹/L)	12.69 ± 7.92	12.23 ± 5.47	0.601
**Neutrophils (%)**	**75.73 ± 14.86 (** *n* ** = 54)**	**70.93 ± 18.53 (** *n* ** = 281)**	**0.073**
**Lymphocyte (%)**	**8.00 (IQR 4.65–13.5) (** *n* ** = 54)**	**10.00 (IQR 6–17) (** *n* ** = 281)**	**0.006**
**Platelet Count (×10³/µL)**	**210.72 ± 123.40**	**273.00 ± 124.13 (** *n* ** = 281)**	**0.001**
**Albumin (g/dL)**	**2.85 ± 0.56 (** *n* ** = 51)**	**3.59 ± 0.61 (** *n* ** = 271)**	**< 0.001**
**Urea (mg/dL)**	**38.00 (IQR 26.5–70.5)**	**30.00 (IQR 18–51) (** *n* ** = 277)**	**< 0.001**
**Creatinine (mg/dL)**	**1.18 (IQR 0.85–2.54)**	**0.93 (IQR 0.725–1.45) (** *n* ** = 280)**	**< 0.001**
**Total Bilirubin (mg/dL)**	**1.20 (IQR 0.78–2.7) (** *n* ** = 54)**	**0.65 (IQR 0.425–1.1) (** *n* ** = 272)**	**< 0.001**
**AST (U/L)**	**92.00 (IQR 49–225) (** *n* ** = 53)**	**27.00 (IQR 18.5–41.5) (** *n* ** = 276)**	**< 0.001**
**ALT (U/L)**	**62.00 (IQR 41.5–99) (** *n* ** = 54)**	**27.00 (IQR 18.5–41) (** *n* ** = 276)**	**< 0.001**
**CRP (mg/L)**	**264.77 (IQR 183.8–316) (** *n* ** = 32)**	**112.54 (IQR 40.5–211.6) (** *n* ** = 112)**	**< 0.001**
**Bacteremia**	**44/55 (80.0%)**	**33/255 (12.9%)**	**< 0.001**
**Bilateral infiltrates**	**28/53 (52.8%)**	**79/281 (28.1%)**	**< 0.001**
**14-day mortality**	**19/55 (34.5%)**	**9/282 (3.2%)**	**< 0.001**

Data are presented as n/N (%) for categorical variables, mean ± standard deviation (SD) for normally distributed continuous variables, and median (interquartile range [IQR]) for non-normally distributed continuous variables. *P-*values are based on the Pearson Chi-square test for categorical variables, the independent samples t-test for normally distributed continuous variables, and the Mann-Whitney U test for skewed continuous variables

Abbreviations: ALT– alanine aminotransferase; AST– aspartate aminotransferase; CLD– chronic liver disease; CKD– chronic kidney disease; CRP– C-reactive protein; DM– diabetes mellitus; HIV– human immunodeficiency virus; IQR– interquartile range; SD– standard deviation

The following variables were considered for inclusion in the multivariable logistic regression model: presentation during the monsoon season, gender, age, presence of diabetes mellitus, icterus, lymphadenopathy, shock, altered sensorium, extrapulmonary focal complications, lymphopenia, thrombocytopenia, raised creatinine, hypoalbuminemia, and bilateral infiltrates. Using a stepwise exclusion approach to minimise redundancy and optimise model performance, four variables remained in the final model. This logistic regression model demonstrated strong discriminatory power for predicting melioidosis in patients with CAP, with a Nagelkerke R² of 0.627 and good calibration (Hosmer-Lemeshow *p* = 0.621). The overall classification accuracy was 91.6%. The four independent predictors retained in the final model were monsoon season exposure (adjusted odds ratio [aOR] = 9.0, 95% CI: 3.6–22.6), diabetes mellitus (aOR = 10.1, 95% CI: 3.6–28.5), shock at presentation (aOR = 17.2, 95% CI: 5.9–49.9), and extrapulmonary focal involvement (aOR = 36.5, 95% CI: 11.0–121.4) (Table 2).

**Table 2:** Logistic regression model to predict melioidosis in patients with CAP

Variables	Unadjusted OR	Adjusted OR	LN aOR	Scores assigned	*p*-value
Presentation in the Monsoon season	8.9 (95%CI: 4.6–17.2)	9.02 (95%CI: 3.6–22.6)	2.200	2	< 0.001
Diabetes	8.4 (95%CI: 3.9–17.8)	10.1(95%CI:3.6–28.5)	2.317	2	< 0.001
Shock at presentation	14.8 (95%CI: 6.9–31.6)	17.1 (95%CI: 5.9–49.9)	2.842	3	< 0.001
Extrapulmonary focal abscesses	14.1 (95%CI: 6.3–31.8)	36.5 (95%CI: 10.9-121.4)	3.596	4	< 0.001

Abbreviations: OR– odds ratio; aOR– adjusted odds ratio; LN aOR– natural logarithm of adjusted odds ratio; CI– confidence interval; CAP– community-acquired pneumonia

**Fig. 2:**
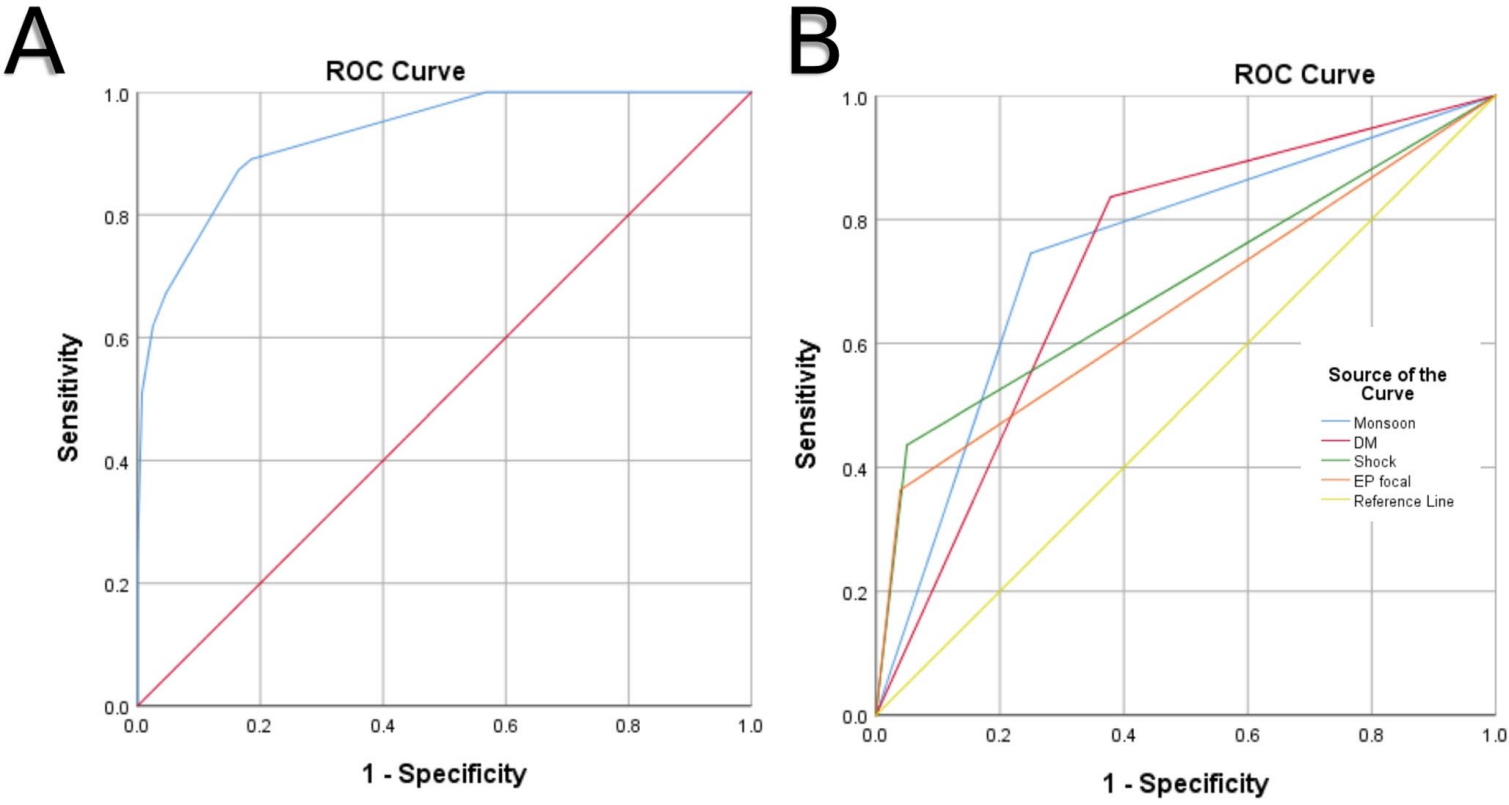
Receiver operating characteristic curve for the scoring system (Panel **A**) and the individual components (Panel **B**) to predict melioidosis in patients with community-acquired pneumonia

Complete data for all four variables were available in 335 patients. The ROC for the scoring system demonstrated excellent diagnostic performance, with an area under the curve (AUC) of 0.930 (95% CI: 0.895–0.965, *p* < 0.001) (Fig. 2A). The maximum possible score was 11. A score of ≥ 4 yielded a sensitivity of 87.3% and specificity of 83.6%, while increasing the threshold to ≥ 5 improved specificity to 95.4% but reduced sensitivity to 67.3%.

Among the individual components of the scoring system, diabetes mellitus showed the highest sensitivity (83.6%) but a modest specificity (62.1%), with an AUC of 0.729 (Fig. 2B; Table 3). Extrapulmonary focal abscesses and shock at presentation demonstrated high specificity (96.1% and 95.0%, respectively), resulting in strong LR⁺ values of 9.33 and 8.72. Monsoon season had a balanced performance, with a sensitivity of 74.5% and specificity of 75.0% (AUC 0.748).

**Table 3:** Diagnostic accuracy and likelihood ratios of individual components of the scoring system

Predictor	AUC	*p*-value	95% CI (Lower–Upper)	Sensitivity	Specificity	LR⁺	LR⁻
Presentation in Monsoon	0.748	< 0.001	0.675–0.820	0.745	0.750	2.98	0.34
Diabetes	0.729	< 0.001	0.661–0.797	0.836	0.621	2.21	0.26
Shock at presentation	0.693	< 0.001	0.605–0.781	0.436	0.950	8.72	0.59
Extrapulmonary focal abscesses	0.662	< 0.001	0.572–0.752	0.364	0.961	9.33	0.66

Abbreviations: AUC– area under the curve; CI– confidence interval; LR⁺– positive likelihood ratio; LR⁻– negative likelihood ratio; DM– diabetes mellitus; ROC– receiver operating characteristic

The distribution of scoring variables differed significantly across pathogens, with melioidosis showing the highest rates of diabetes, shock, monsoon presentation, and extrapulmonary abscesses (Table 4).

**Table 4:** Comparison of key clinical variables across different microbial etiologies of community-acquired pneumonia

Variable	Melioidosis	Pneumococcus	Haemophilus influenzae	Klebsiella pneumoniae	Escherichia coli	Staphylococcus aureus	*p*-value
Diabetes	46/55 (83.6%)	22/86 (25.6%)	16/44 (36.4%)	52/103 (50.5%)	6/13 (46.2%)	10/34 (29.4%)	< 0.001
Shock	24/55 (43.6%)	7/86 (8.1%)	0/44 (0.0%)	4/105 (3.8%)	2/13 (15.4%)	1/34 (2.9%)	< 0.001
Monsoon Presentation	41/55 (74.5%)	22/86 (25.6%)	8/44 (18.2%)	29/105 (27.6%)	2/13 (15.4%)	9/34 (26.5%)	< 0.001
Extrapulmonary Focal Abscess	20/55 (36.4%)	4/86 (4.7%)	0/44 (0.0%)	5/105 (4.8%)	0/13 (0.0%)	2/34 (5.9%)	< 0.001

## Discussion

This retrospective study analysed 337 cases of CAP, including 55 confirmed cases of melioidosis, to identify distinguishing clinical features and derive a diagnostic tool to aid early recognition. Melioidosis cases were significantly associated with higher disease severity, increased 14-day mortality, and a unique epidemiological and clinical profile. Compared to controls with other confirmed bacterial pneumonias, melioidosis patients more frequently presented during the monsoon season, were older, predominantly male, and had a high prevalence of diabetes mellitus. They also exhibited more systemic involvement, including shock, altered sensorium, and extrapulmonary focal complications.

Univariate analysis identified several factors significantly associated with melioidosis. Demographically, male gender, older age, and diabetes mellitus were overrepresented, consistent with known risk groups for *Burkholderia pseudomallei*. Most studies show a high prevalence of diabetes in patients with melioidosis [8, 12, 13]. In a systematic review and meta-analysis, it was noted that close to half of the patients with melioidosis have diabetes, and diabetic patients are three times more likely to have melioidosis [14]. This finding is especially relevant, considering the fact that between 1990 and 2022, South Asia, in comparison to other regions, experienced one of the most significant increases in diabetes prevalence, especially in Pakistan, while treatment coverage remained low [15]. Diabetes predisposes individuals to melioidosis by impairing both innate and adaptive immunity [16]. Hyperglycemia disrupts neutrophil function, reduces pro-inflammatory cytokine production, and alters macrophage and T cell responses [16]. These immune deficits compromise the host’s ability to contain *Burkholderia pseudomallei*, resulting in increased susceptibility and more severe clinical manifestations [16]. Most study cohorts show male predominance and a median age of over 50 years [8, 12, 13]. Males over 50 years are more likely to engage in outdoor activities such as farming or construction, which increase contact with contaminated soil or water. Additionally, age-related immune changes and a higher prevalence of comorbidities like diabetes in this demographic may further elevate susceptibility to *Burkholderia pseudomallei* infection. It should also be noted that, in general, sepsis-related hospitalisation due to any aetiology is more common in older men compared to women [17].

In addition to host-related factors, studies report a significant increase in cases during the rainy season and a strong positive correlation between monthly precipitation and melioidosis incidence [13]. Melioidosis incidence strongly correlates with climatic factors, particularly high maximum rainfall and dense cloud cover, which create moist soil conditions ideal for *Burkholderia pseudomallei* survival [18]. In a study that examined seasonal trends in melioidosis cases over 10 years in southern India, 77.4% of cases occurred during the wet season (May–December), with a peak in the monsoon months [19]. In a time-series analysis from Australia, intense rainfall and cloud cover were strong predictors of melioidosis [20]. Additionally, tropical cyclones were temporally associated with clusters of melioidosis cases [20]. These findings align closely with our results, reinforcing the inclusion of seasonality as a key variable in our predictive model.

Clinically, features such as shock, icterus, lymphadenopathy, altered sensorium, and extrapulmonary involvement in our cohort reflect the virulence of *B. pseudomallei*, which has been consistently reported in other endemic settings. The Darwin prospective study noted that 20% of melioidosis patients developed septic shock, and multisystem involvement was frequently observed, particularly in individuals with diabetes [8]. Icterus and elevated transaminases, indicative of hepatic involvement, were also commonly reported in these studies, supporting our findings of elevated liver enzymes and bilirubin [8, 13]. Laboratory abnormalities observed in our cohort, such as lymphopenia, thrombocytopenia, hypoalbuminemia, and elevated creatinine, are consistent with the systemic inflammatory response and multi-organ dysfunction characteristic of severe melioidosis [8, 13]. Extrapulmonary focal complications were significantly more frequent among melioidosis cases in our cohort and served as one of the strongest independent predictors in the final logistic regression model. Melioidosis is well recognised for its propensity to cause abscess formation in a wide range of organs, including the liver, spleen, prostate, kidneys, and soft tissues [21]. These focal infections are often insidious and may be overlooked without systematic imaging of internal organs. In the Darwin prospective study, 46% of patients had at least one internal organ abscess [8]. Similarly, other studies have reported a high propensity for abscesses in soft tissue, bones and internal organs [12, 13]. These manifestations reflect the organism’s ability to disseminate hematogenously and establish a persistent localised infection. Consistent with this, bacteremia was significantly more common among patients with melioidosis. In contrast, control pathogens such as *Streptococcus pneumoniae* may have been more susceptible to empiric antibiotic therapy, which might have been initiated before blood cultures were obtained.

In resource-limited settings where advanced imaging may not be routinely available, clinical signs such as localised pain, tenderness, or swelling, particularly in patients with diabetes, should raise suspicion for melioidosis. Liver, spleen, and kidney abscesses, characteristic of disseminated melioidosis, can often be identified using point-of-care ultrasound (POCUS), a practical and rapid bedside tool. These focal complications are frequently insidious and may be missed without targeted imaging. Incorporating POCUS into the initial evaluation of patients presenting with sepsis and respiratory symptoms can help detect these hallmark features early. In recent years, POCUS has seen increasing adoption in India, particularly in tertiary care hospitals, academic centres, and urban emergency departments. While its availability at the primary health care level remains limited, several district hospitals and secondary-level facilities have begun integrating POCUS into routine diagnostic workflows, particularly for trauma, obstetric emergencies, and sepsis evaluation. Given its portability, ease of use, and diagnostic value, there is a strong case for wider advocacy and training in POCUS as a critical tool for early detection of extrapulmonary complications in resource-limited settings. Nevertheless, we acknowledge that its access is not yet universal, and future studies should explore clinical examination alone to identify extrapulmonary focal abscesses.

In multivariable logistic regression, four independent predictors emerged: monsoon season, diabetes mellitus, shock at presentation, and extrapulmonary focal involvement. These variables demonstrated strong associations with melioidosis and retained statistical significance after adjusting for confounders. Their biological plausibility, ease of clinical assessment, and limited overlap with other bacterial pneumonias make them practical markers for early identification. A simple, evidence-informed scoring system was developed based on the adjusted odds ratios of these four predictors. Each variable was assigned a score based on its logarithmic odds ratio, creating a tool with a maximum score of 11. This model showed excellent diagnostic performance, with an AUC of 0.930. A cut-off score of ≥ 4 achieved a balance of high sensitivity (87.3%) and specificity (83.6%), while a cut-off of ≥ 5 provided excellent specificity (95.4%) with moderate sensitivity. A cut-off score of ≥ 4 might be preferred for initial screening due to its higher sensitivity, especially in primary care or low-resource settings. A score of ≥ 5 may be more appropriate where specificity and antibiotic stewardship are prioritised. Using only four readily assessable clinical features, the tool’s simplicity allows it to be used effectively at the primary care level, especially in resource-limited settings where laboratory support may be unavailable. Variables with high specificity, such as extrapulmonary focal abscesses and shock, are particularly useful for ruling in melioidosis (LR⁺ >8). In contrast, predictors like diabetes and monsoon season offer greater sensitivity, aiding initial clinical suspicion. When individual organisms were compared, melioidosis showed a distinct clinical profile. While *Klebsiella pneumoniae* and *Escherichia coli* shared some features, such as diabetes and shock, with melioidosis, their prevalence was significantly higher in melioidosis, reinforcing the discriminative power of the selected predictors in the scoring system.

To support real-world utility, prospective validation of this scoring system is essential across diverse clinical settings, particularly in endemic regions with limited diagnostic resources. Future studies should aim to implement the score in district hospitals, emergency departments, and primary health centres where microbiological facilities may be limited or delayed. Incorporating the score into triage protocols could facilitate early identification of high-risk patients, prompt empirical initiation of appropriate antibiotics, and guide referrals. Additionally, community health workers or mobile clinics equipped with POCUS could apply the score in remote areas. Validation studies should also assess interobserver reliability, ease of use in resource-constrained settings, and clinical impact on antibiotic selection and patient outcomes. This would help determine whether simplified or modified versions, such as those excluding POCUS, are necessary in settings where imaging access remains limited.

While the implementation of such a scoring tool may lead to overidentification in some cases, resulting in the empiric use of higher-end antibiotics like ceftazidime or meropenem, this trade-off is justifiable given the high mortality and severe disease course associated with untreated melioidosis. Early and appropriate empirical therapy is critical in improving outcomes, particularly when culture confirmation is delayed or not feasible. In our study, melioidosis cases were associated with significantly higher 14-day mortality and systemic involvement, underscoring the importance of timely recognition. The benefits of early treatment likely outweigh the risks of overtreatment in this context, especially when applied to high-risk patients with classic epidemiological and clinical features. Moreover, the focused inclusion of four clinically assessable predictors enhances specificity and helps minimise unnecessary broad-spectrum antimicrobial use. This approach offers a pragmatic balance between diagnostic sensitivity and antimicrobial stewardship in endemic regions.

Several limitations must be acknowledged. The retrospective design introduces potential bias due to missing data and unmeasured confounders. Important epidemiological risk factors such as occupation, environmental exposures, and place of residence could not be reliably assessed. Our study focused exclusively on patients with acute CAP, defined as a symptom duration of 14 days or less. This threshold was applied to ensure alignment with established case definitions and to facilitate meaningful comparison across patients presenting with acute illness. While melioidosis can present in subacute or chronic forms, we specifically aimed to identify predictors in those presenting acutely, where timely recognition is critical and empiric therapy often needs to be initiated before microbiological confirmation. We acknowledge that by excluding subacute presentations, a subset of melioidosis cases may have been omitted. Similarly, we excluded patients with pulmonary tuberculosis (TB) due to its typically subacute-to-chronic presentation. Although TB can clinically mimic melioidosis and is an important differential diagnosis in endemic regions, true acute TB presentations within 14 days are uncommon. Including TB cases would have introduced heterogeneity into our cohort and potentially reduced the internal validity of our scoring model. Nevertheless, we recognise that in real-world clinical settings, diagnostic confusion between TB and melioidosis is common, and this remains an important consideration for future prospective validation efforts. The study relied exclusively on culture-confirmed cases of CAP, which may underestimate the burden of melioidosis while enhancing diagnostic specificity due to the limited sensitivity of bacterial cultures and potential underdiagnosis. Patients with atypical pneumonia or undifferentiated tropical febrile illnesses were omitted. Moreover, although we applied standardised definitions for CAP, our requirement for radiological infiltrates may have led to the exclusion of cases with subtle or early lung involvement, particularly given the limited sensitivity of chest X-rays. Our analysis also focused solely on patients with bacteriologically documented CAP, representing only a subset of all CAP presentations. The greater diagnostic challenge in clinical practice lies in evaluating patients with culture-negative pneumonia. As such, the performance of our scoring system in this broader and more representative population remains uncertain. Including a random sample of non-culture-confirmed CAP cases in future validation studies would help assess whether their clinical profiles align with the bacteriologically confirmed cohort and determine if model recalibration is necessary. Another limitation of our study is the exclusion of patients with polymicrobial growth in sputum cultures. This decision was taken to ensure clear attribution of clinical features and outcomes to a single causative organism, which was necessary for the development of a robust and interpretable scoring system. However, we acknowledge that coinfections are commonly encountered in routine clinical care, and the applicability of our scoring system in the context of polymicrobial infections remains uncertain. Additionally, this was a single-centre study conducted in a tertiary care hospital in southern India, which may limit generalizability to other geographic settings with different endemic patterns, healthcare access, and population characteristics.

In conclusion, melioidosis is an important but often overlooked cause of CAP in endemic regions, particularly among diabetic patients during the monsoon season. The proposed four-variable scoring system provides a pragmatic and clinically intuitive tool for early recognition and targeted management. After external and prospective validation, it holds promise for reducing diagnostic delays, optimising antimicrobial use, and improving outcomes, particularly in settings where diagnostic resources are limited and clinical uncertainty is high. 

## Electronic supplementary material

Below is the link to the electronic supplementary material.

Supplementary Material 1

## Data Availability

Data Availability Statement: The datasets generated and/or analysed during the current study are not publicly available due to patient confidentiality and institutional policies, but are available from the corresponding author (NG) upon reasonable request and with appropriate institutional approvals.
