# Supplementary material for: Clinical scoring system to differentiate melioidosis from other documented causes of community-acquired bacterial pneumonia: a retrospective cohort study
Source: Infection. 2025 Jul 23;53(6):2659–68. doi: 10.1007/s15010-025-02611-y (PMC12675767; doi:10.1007/s15010-025-02611-y)
Supplement: Supplementary file 1 — Supplementary Material 1 [file 15010_2025_2611_MOESM1_ESM.docx]

**Supplementary Tables**

Supplementary Table 1: Inclusion and Exclusion Criteria for Study Participants

| **Criterion** | **Included Patients** | **Excluded Patients** |
| --- | --- | --- |
| Age | ≥18 years | <18 years |
| Chest X-ray (CXR) infiltrate | Present (new-onset radiological infiltrate) | Absent |
| Symptom duration | ≤14 days | >14 days |
| Core clinical features | At least two of: fever ≥38°C, cough, or dyspnoea | Fewer than two of the above, or not documented |
| Microbiological confirmation | Bacterial culture positivity from respiratory specimen or blood within 2 days of admission | No culture positivity or culture after 48 hours of admission |
| Infection setting | Community-acquired | Hospital-acquired (hospitalisation within prior 14 days or culture >48 hours after admission) |
| Causative organism | Common CAP pathogens | Organisms uncommon in CAP (e.g., Pseudomonas aeruginosa, Acinetobacter spp., Stenotrophomonas maltophilia) |
| Sputum culture | Single organism isolated | Polymicrobial growth in the same sample |

Supplementary Table 2: Definitions of Demographics, Clinical and Laboratory Variables

| **Variable** | **Definition / Description** |
| --- | --- |
| **Monsoon Season** | June to September |
| **Fever** | Axillary/body temperature ≥38.0°C |
| **Dyspnoea** | Self-reported or clinically documented respiratory distress |
| **Shock** | Systolic BP <90 mmHg or MAP <65 mmHg, requiring vasopressors |
| **Altered sensorium** | Any change in consciousness, awareness, or orientation from baseline |
| **Icterus** | Clinically visible yellowing of sclera or skin |
| **Lymphadenopathy** | Palpable enlargement of lymph nodes |
| **Neck stiffness** | Resistance to passive neck flexion on clinical examination |
| **Extrapulmonary abscess** | Abscesses in intra-abdominal, musculoskeletal, or soft tissues (clinical or ultrasound, including point-of care ultrasound) |
| **Lymphopenia** | Absolute lymphocyte count <1100 cells/mm³ |
| **Thrombocytopenia** | Platelet count <150,000 cells/μL |
| **Raised creatinine** | Serum creatinine >1.2 mg/dL at presentation |
| **Hypoalbuminemia** | Serum albumin <3.5 g/dL |
| **Raised transaminases** | AST or ALT values above institutional upper limit of normal |
| **Radiological findings** | Presence of cavitary lesions, unilateral or bilateral infiltrates on chest X-ray |
| **Diabetes mellitus** | Pre-existing diagnosis or newly diagnosed with HbA1c ≥6.5% |
